# DORSSAA: Drug-Target interactOmics Resource Based on Stability/Solubility Alteration Assay

**DOI:** 10.1016/j.mcpro.2026.101603

**Published:** 2026-06-17

**Authors:** Ehsan Zangene, Elham Gholizadeh, Veit Schwämmle, Amir Ata Saei, Mathias Wilhelm, Lukas Käll, Mohieddin Jafari

**Affiliations:** 1 Department of Pharmacology, University of Helsinki, Helsinki, Finland; 2 Department of Biochemistry and Molecular Biology, University of Southern Denmark, Odense, Denmark; 3 Department of Microbiology, Tumor and Cell Biology at Karolinska Institutet, Stockholm, Sweden; 4 Computational Mass Spectrometry, TUM School of Life Sciences, Technical University of Munich, Freising, Germany; 5 Munich Data Science Institute, Technical University of Munich, Garching, Germany; 6 Science for Life Laboratory, School of Engineering Sciences in Chemistry, Biotechnology and Health, KTH Royal Institute of Technology, Stockholm, Sweden; 7 Faculty of Medicine and Health Technology, Tampere University and TAYS Cancer Center, Tampere, Finland; 8 Tampere Institute for Advanced Study, Tampere University, Tampere, Finland

**Keywords:** drug-target interactions, thermal proteome profiling (TPP), proteome integral solubility alteation (PISA), proteome stability, proteomce solubility, chemical proteomics, drug discovery, target identification, proteomics database, web resource

## Abstract

Advancements in high-throughput techniques such as Thermal Proteome Profiling and the high-throughput Proteome Integral Solubility Alteration assay have revolutionized our understanding of drug-protein interactions. Despite these innovations, the absence of an integrative platform for cross-study analysis of stability and solubility alteration data represents a significant bottleneck. To address this gap, we introduce Drug-target interactOmics Resource based on Stability/Solubility Alteration Assay (DORSSAA), an interactive and expandable web-based platform for the systematic analysis and visualization of proteome stability and solubility alteration assay datasets. Currently, DORSSAA features 1,135,985 records spanning 38 cell lines and organisms, 135 compounds, and 40,742 protein targets. Through its user-friendly interface, the resource supports comparative drug-protein interaction analysis and facilitates the discovery of actionable therapeutic targets. Through two case studies, methotrexate target profiling in A549 cells and combinatorial-therapy drug-target interactions in leukemia cell lines, we demonstrate DORSSAA’s utility for identifying protein-drug interactions across diverse experimental contexts. This resource empowers researchers to accelerate drug discovery and enhance our understanding of protein behavior. Compared with data repositories and interaction databases, DORSSAA provides direct protein-level evidence of mechanisms of action with strict statistical control for each study. This enables more reliable identification of drug targets, off-target effects, and potential drug combinations.

## Highlights

• DORSSAA integrates TPP and PISA drug–protein interaction datasets.
• Contains 1.1 M + records across 38 cell lines and organisms.
• Enables comparative analysis of stability and solubility alterations.
• Supports discovery of drug targets, off-targets, and combinations.
• Case studies validate utility across diverse experimental contexts.

## Graphical abstract

**Figure:**
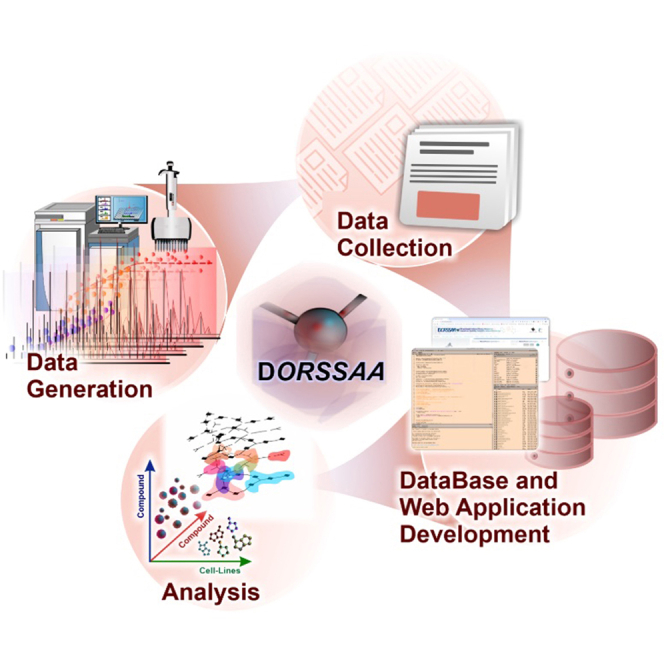

## In Brief

DORSSAA is a web-based resource for systematic analysis of drug–protein interactions derived from thermal stability and solubility alteration assays, including TPP and PISA. By integrating over one million records across diverse compounds, cell lines, and organisms, the platform enables comparative exploration of protein-level drug responses, target identification, and off-target analysis. Case studies demonstrate its utility for mechanism-of-action studies and combinatorial drug discovery across different biological contexts.

Drug discovery is a multidisciplinary field encompassing medicine, biotechnology, and pharmacology, aimed at identifying novel therapeutic compounds. Despite significant advances, a major challenge remains in the precise characterization of drug targets and off-targets within complex cellular environments. This challenge is especially pronounced in oncology, where identifying cancer vulnerabilities and dependencies, such as synthetic lethality relationships, is vital for developing precision therapies (1, 2, 3). This limitation is compounded by the absence of direct, systematic methods to monitor the effects of drugs on proteins within cells and tissues (4). Traditional evaluations by regulatory bodies such as the FDA, including absorption, distribution, metabolism, and excretion studies and animal toxicity assessments, often fall short of fully elucidating a drug's mechanism of action (MoA) (5). From a drug MoA point of view, identifying effective drug targets is challenging in drug development. As a result, many drug candidates fail during late-stage clinical trials (phase II trials), leading to resource wastage and underscoring the urgent need for innovative strategies to enhance drug development (6).

While small molecule modulators hold promise as therapeutic agents, their diverse effects on protein targets necessitate comprehensive profiling to better understand their molecular MoA. Transcriptomic approaches such as RNA sequencing and the Connectivity Map have been instrumental in mapping drug action (7). However, because most FDA-approved drugs target proteins and protein and RNA levels often correlate weakly, protein-level changes are likely to provide the more relevant readout of compound action (8, 9, 10, 11).

Recent innovations in solubility and stability alteration assays have revolutionized the study of drug-protein interactions (12, 13). These techniques, including cellular thermal shift assay, thermal proteome profiling (TPP), and proteome integral solubility alteration (PISA) assay, leverage changes in protein stability or solubility upon ligand binding or environmental perturbations, offering an unbiased, high-throughput approach to probe drug interactions at the proteome level. By subjecting samples to varying conditions, these methods uncover critical insights into protein behavior and drug interactions across diverse biological contexts (14, 15). Importantly, the biological interpretation depends strongly on experimental context. When compounds are applied to lysates or extracts, the resulting stability/solubility shifts are often more informative for candidate direct target nomination, whereas intact-cell experiments may additionally capture downstream pathway and MoA-related effects within the cellular environment. Due to its numerous advantages, this approach has become widely used in both drug discovery and chemical proteomic research.

Centralized, queryable proteomics repositories are increasingly critical for nominating novel drug targets and dependencies, and for designing next-generation therapies. By enabling cross-dataset replication and mechanism-anchored readouts at the protein level, such resources help prioritize actionable targets, reveal context-specific vulnerabilities, and move beyond transcript-only inferences. Here, we present Drug-target interactOmics Resource based on Stability Solubility Alteration Assay (DORSSAA); https://dorssaa.it.helsinki.fi/), a dedicated resource for the systematic analysis and visualization of drug-associated solubility and stability alteration datasets from TPP/PISA-family experiments. DORSSAA integrates high-throughput experimental data encompassing 1,135,985 records from 38 cell lines and organisms, 135 compounds, and 40,742 protein targets, providing an unprecedented dataset for drug interactomics. By consolidating this data into an intuitive, user-friendly web application, DORSSAA enables researchers to explore drug-target interactions across diverse experimental conditions. This resource helps validate assay-based findings and supports context-specific exploration of potential drug–protein interactions in TPP/PISA-type assays.

General proteomics repositories (*e.g.,* PRIDE/ProteomeXchange) host raw and processed MS-based proteomics but do not harmonize thermal shift/solubility readouts into queryable, per-protein effect sizes across compounds and cell lines (16). Drug-target knowledgebases (*e.g.,* DrugBank, ChEMBL, BindingDB) curate biochemical/affinity interactions, rather than proteome-wide stability/solubility shifts (17). Perturbational resources (*e.g.,* LINCS/Connectivity Map) profile transcriptomic responses (L1000) instead of protein stability/solubility (7). Drug combination portals (*e.g.,* DrugComb) focus on sensitivity/synergy metrics and do not provide protein-level thermal/solubility shift evidence (18). Meltome Atlas is a valuable complementary resource for baseline thermal stability landscapes across species and biological contexts, but it is not a perturbation-focused drug-target interactomics database and does not catalog compound-induced stability or solubility shifts. Likewise, DTC, MICHA, and Drug Target Interact provide curated drug-target knowledge, compound annotations, or external validation layers, but they do not function as assay-native repositories of proteome-wide thermal/solubility-shift signals measured directly in defined biological contexts. Thus, unlike these resources, DORSSAA is specifically centered on experimentally observed compound-induced thermal/solubility shifts at the proteome level in defined biological contexts. DORSSAA offers several key advantages that make it a valuable complement to data repositories and interaction knowledgebases. It organizes assay-native, protein-level outputs from TPP, PISA, i-PISA, and CoPISA studies into a unified relational schema for structured querying, visualization, and cross-study exploration while preserving assay and study metadata for contextual interpretation. Its statistical framework standardizes significance-related fields through empirical DMSO-null *p*-values for selected PISA datasets, per-study false discovery rate control via Storey q-values with BY fallback where needed, and safe *p*-value handling. However, this harmonization is intended to support discoverability and comparative exploration, not to claim full quantitative equivalence across fundamentally different assay types. DORSSAA also supports rich context modeling, incorporating cell-line and organism metadata, concentration and replicate tracking, and providing volcano and coverage views. Furthermore, it includes similarity layers, such as chemotype and cell-line similarity, to help generalize hit findings. Finally, CoPISA models evidence from drug combinations in a way that supports more rational selection of drug pairs (3, 19) beyond traditional synergy scores. In the current version, DORSSAA intentionally focuses on TPP/PISA-family assays because these methods share a related biophysical readout, currently represent the most mature and publicly available body of stability/solubility-shift datasets, and can therefore be harmonized within a common schema. We acknowledge that other chemoproteomic approaches, including activity-based protein profiling, kinase pulldown methods, LiP-MS, PELSA, pulse proteolysis, and DARTS, can also provide valuable candidate drug-target information; however, incorporating such orthogonal modalities would require expansion of the evidence model and is an important future direction for DORSSAA.

## Experimental Procedures

### Collection of Proteomics Data

We assembled a harmonized corpus of 24 publicly available thermal-shift proteomics datasets, covering TPP, PISA, and i-PISA, together with our in-house CoPISA dataset (20, 20, 21). In total, the resource spans 135 small molecules profiled across 38 cancer cell lines or species. Each study was reshaped to a tidy long format and enriched with consistent metadata (PMID, compound, cell line, UniProt ID, gene symbol, replicate, concentration, effect size, and statistical fields). Importantly, experimental context, including whether compound treatment was performed in intact cells or in lysate/extract preparations, was preserved as study-level metadata because these settings support different biological interpretations and should not be treated as interchangeable evidence types. Compound names were linked to PubChem InChIKeys, and cell lines were annotated with Cellosaurus and organism information. We then generated a coverage map (cell line × compound) and a normalized interaction fact table that captures per-protein stabilization/destabilization signals and their significance, and materialized everything into an indexed SQL backend for fast, app-ready queries in DORSSAA.

We curated a representative corpus of thermal-shift proteomics studies across assays, models, and temperature regimens (Table 1), which DORSSAA harmonizes for cross-study exploration.

**Table 1:** Overview of studies included in DORSSAA, showing assay type, treatment context (intact cells or extracts/lysates), biological model or sample preparation, and temperature regime

No.	Study title	Assay	Model and treatment context; temperature (°C)	Reference
1	Shifting Beyond Classical Drug Synergy in Combinatorial Therapy through Solubility Alterations	CoPISA	SKM-1, NOMO-1, MOLM-13, MOLM-16 (intact & extract); 48–59 °C	(21)
2	The antimicrobial drug pyrimethamine inhibits STAT3 transcriptional activity by targeting the enzyme dihydrofolate reductase	PISA	U3A (intact); 43–57 °C	(22)
3	Thermal proteome profiling for unbiased identification of direct and indirect drug targets using multiplexed quantitative mass spectrometry	TPP	K562 (intact); 37–67 °C	(23)
4	Lysine-specific demethylase 1A restricts *ex vivo* propagation of human HSCs and is a target of UM171	TPP	HL-60 (intact); 37–67 °C	(24)
5	Thermal Proteome Profiling Identifies Oxidative-Dependent Inhibition of the Transcription of Major Oncogenes as a New Therapeutic Mechanism for Select Anticancer Compounds	TPP	MCF7 (intact); 37–67 °C	(25)
6	Anticancer Effect of Deuterium Depleted Water - Redox Disbalance Leads to Oxidative Stress	TPP	A549 (intact); 37–67 °C	(26)
7	Proteome Integral Solubility Alteration: A High-Throughput Proteomics Assay for Target Deconvolution	PISA	A549 (intact & extract); 43, 44.7, 46.4, 48.1, 49.8, 51.5, 53.2, 54.9, 56.6, 58 °C	(14)
8	Ion-Based Proteome-Integrated Solubility Alteration Assays for Systemwide Profiling of Protein-Molecule Interactions	I-PISA	A549, K562 (intact & extract); 37, 42, 46, 50, 54, 58, 62, 67 °C	(27)
9	A Simplified Thermal Proteome Profiling Approach to Screen Protein Targets of a Ligand	STPP	293T (extract); 53–56, 66, 69, 72 °C	(28)
10	Thermal Proteome Profiling Reveals Glutathione Peroxidase 4 as the Target of the Autophagy Inducer Conophylline	TPP	U-2 OS (extract); 40, 43, 46, 49, 52, 55, 58, 61, 64, 67 °C	(29)
11	Thermal proteome profiling identifies PIP4K2A and ZADH2 as off-targets of Polo-like kinase 1 inhibitor volasertib	TPP	Jurkat (intact); 37, 41, 44, 47, 50, 53, 56, 59, 63, 67 °C	(30)
12	Thermal proteome profiling monitors ligand interactions with cellular membrane proteins	TPP	K562 (ATP), Jurkat (pervanadate) (intact & extract); 37–67 °C	(31)
13	Proteome-wide solubility and thermal stability profiling reveal distinct regulatory roles for ATP	TPP	Jurkat E6.1 (extract); 37–66.3 °C	(32)
14	Cell surface thermal proteome profiling tracks perturbations and drug targets on the plasma membrane	TPP	K562 (intact); 37–67 °C	(33)
15	Identifying the Target of an Antiparasitic Compound in Toxoplasma Using Thermal Proteome Profiling	TPP	Toxoplasma (CDPK1G, CDPK1M) (intact); 37–67 °C	(34)
16	Thermal proteome profiling of breast cancer cells reveals proteasomal activation by CDK4/6 inhibitor palbociclib	TPP	MCF7 (intact); 37–65 °C	(35)
17	Precipitate-Supported Thermal Proteome Profiling Coupled with Deep Learning for Comprehensive Screening of Drug Target Proteins	PSTPP	HEK293 T, K562 (extract); 44, 52, 53, 65 °C	(36)
18	Thermal proteome profiling allows quantitative assessment of interactions between tetrachloroethene reductive dehalogenase and trichloroethene	TPP	*Sulfurospirillum multivorans* (extract); 43–97 °C	(37)
19	Thermal Proteome Profiling in Zebrafish Reveals Effects of Napabucasin on Retinoic Acid Metabolism	TPP	Zebrafish embryo (extract); 34–64 °C	(38)
20	Tracking cancer drugs in living cells by thermal profiling of the proteome	TPP	K562 (intact & extract); 37–67 °C	(12)
21	Systematic analysis of chemical-protein interactions from zebrafish embryo by proteome-wide thermal shift assay, bridging the gap between molecular interactions and toxicity pathways	PISA	Zebrafish embryo (extract); 37–67 °C	(39)
22	Identification of Celecoxib-Targeted Proteins Using Label-Free Thermal Proteome Profiling on Rat Hippocampus	TPP	Rat (extract); 67 °C	(13)
23	Network integration of thermal proteome profiling with multi-omics data decodes PARP inhibition	2D-TPP	UWB1.289 (intact); 42.1, 44.1, 46.2, 48.1, 50.4, 51.9, 54, 56.1, 58.2, 60.1, 62.4, 63.9 °C	(40)
24	Large-scale characterization of drug mechanism of action using proteome-wide thermal shift assays	PISA	K562 (intact & extract); 48–58 °C	(41)

TPP, Thermal Proteome Profiling; PISA, proteome integral solubility alteration.

**Table 2:** Database tables in DORSSAA, record counts, and where they appear in the app

Table name	# Records	Description (content & data types)	Shown in app tabs
Cell line	38	Unique cell lines with identifiers (character), Cellosaurus IDs (character), organism (factor/chr)	*protein/Cell-Line/compound tabs*
Compounds	135	Unique compounds (character), PubChem InChIKey (character)	*protein/Cell-Line/compound tabs*
DTC Target	480,456	Drug-target-common records use here as an external validation	*protein/Cell-Line/compound tabs*
Interaction	1,135,985	Fact table of protein-compound effects: UniProt, gene symbol, cell line, PMID, log2FC, p/FDR, dose	*protein/Cell-Line/compound tabs*
Target	40,742	Unique protein targets: UniProt ID, gene symbol	*protein/Cell-Line/compound tabs*
Cell lines similarity	55	Showing how different cell lines are similar based on different expressed biomarkers	*Cell-line tab*
Compound similarity	528	Pairwise compound similarity from SMILES fingerprints (drug1, drug2, similarity; numeric/char)	*Compound tab*
Drug target interaction	19,378	Curated drug-target interactions records use here as an external validation	*protein/Cell-Line/compound tabs*
Meltome	1,858,170	Meltome/thermal stability records (species/protein) for each protein of interest	*protein/Cell-Line/compound tabs*
MICHA	367,628	Micha compound annotations (IDs, primary targets; character)	*protein/Cell-Line/compound tabs*

### Data Normalization and Handling Missing Values

The goal of harmonization was to standardize heterogeneous study outputs into a common statistical representation suitable for querying, filtering, and cautious cross-study exploration, while preserving the original assay type, study provenance, and experimental context. Accordingly, we did not treat these steps as establishing full statistical equivalence across assays; rather, they were designed to improve the interpretability of reported evidence fields across studies.

To ensure cross-study consistency, we standardized column names (replacing non-word separators with underscores), reconciled identifiers (prioritizing UniProt IDs and mapping HGNC symbols to UniProt via biomaRt when needed), and split multi-gene or multi-UniProt entries into separate rows. Inferred UniProt IDs are explicitly marked with a cloud symbol (☁) for transparency (42).

Reported significance values were represented on a common scale wherever possible. Specifically, reported −log10(p) values were converted back to raw *p*-values when needed so that significance fields from different studies could be queried and filtered consistently. Extreme or zero values were then safely clamped to avoid numerical artifacts caused by rounding, truncation, or underflow during downstream correction procedures. Multiple testing was controlled within study-defined families, specifically per (PMID × compound), to preserve the original experimental context rather than combining unrelated studies into a single correction framework. We did not apply a single cumulative or global FDR correction across all datasets in DORSSAA, because the resource integrates heterogeneous processed study outputs rather than pooling raw proteomics evidence into one unified discovery analysis. We used Storey’s q-value approach with robust π_0_ estimation when feasible, and automatically fell back to the more conservative Benjamini–Yekutieli procedure for small or problematic families. For the K562 PISA study, we used an empirical DMSO-based null framework, together with light row-mean imputation only when missingness was limited (43), two-sided scoring with add-one smoothing, and per-drug Benjamini–Hochberg FDR control, because this dataset required a study-specific significance strategy matched to its design. Replicate structure was preserved through dedicated replicate metadata and replicate-correlation QC, and variability across studies is visualized in DORSSAA via volcano plots so users can judge effect sizes in their experimental context. Importantly, these processing steps standardize identifiers, metadata, and statistical fields for unified querying and filtering, but they do not force direct quantitative equivalence across assay formats. Likewise, DORSSAA does not combine *p*-values from different studies into a single unified inferential statistic. Accordingly, recurring associations observed across studies should not be interpreted as a single globally FDR-controlled discovery list, but rather as context-dependent patterns preserved from study-level evidence. Reported *p*-values are retained as study-specific evidence fields represented on a common scale for database querying, while their interpretation remains tied to the original statistical framework used in each study. Therefore, effect sizes, significance values, and target rankings should be interpreted in light of assay type, sample preparation, experimental design, and the original source study. As a result, cross-study comparisons of *p*-value magnitude or fold-change magnitude should be regarded as descriptive and exploratory unless the underlying studies share sufficiently similar experimental and statistical frameworks. This includes careful separation of lysate/extract-based and intact-cell evidence, because these datasets do not provide identical types of biological information and should not be interpreted as directly interchangeable for target identification.

### Protein Query Database Interface

The Protein Query tab provides a versatile platform for users to investigate compounds treated within specific cellular contexts or organisms, enabling the identification of individual drug-target interactions or comprehensive database exploration. Query outputs are presented in multiple formats, including bar charts, volcano plots, and detailed data tables containing interaction-specific information. Users can further refine these results based on the significance thresholds of reported *p*-values. To improve interpretability, the result tables now also expose treatment context from the underlying study metadata, allowing users to distinguish whether a reported association originated from intact-cell or lysate/extract-based experiments and to search these evidence types separately within the interactive table interface.

Additionally, the interface facilitates cross-referencing of protein identities with UniProt records and provides direct access to external databases, such as DTC (44), MICHA, and Drug Target Interact (45), for extended information on drug-target interactions (44, 46).

### Cell Lines Query Database Interface

The Cell Line Query tab offers a powerful platform for investigating critical biological questions by enabling users to explore the scope and extent of drug-protein interactions across various cellular contexts. By integrating insights from the Protein Query tab, users can evaluate which cancer cell lines or organisms are most conducive to drug-protein interactions and which are less responsive. This functionality provides valuable insights into pan-cancer and cancer-specific interactions, supporting the development of personalized therapeutic strategies. To enhance user experience and analysis capabilities, the interface includes advanced tools and features, such as interaction filtering based on *p*-values, UniProt protein annotations, external checks with UniProt, and cross-references to external drug-target interaction databases. These resources ensure comprehensive exploration and interpretation of interaction data within relevant biological contexts.

Additionally, the interface incorporates a melting points distribution tool sourced from the Meltome database (47). This tool allows users to visualize the melting point diversity of queried proteins across different contexts. Using box plots separated by cell line and a combined box plot for all cell lines, researchers can gain critical insights into melting point variations, providing a valuable perspective when designing experiments.

### Compound Query Database Interface

The Compound Query tab supports reverse protein-to-compound exploration by enabling users to investigate which compounds in DORSSAA are associated with a selected protein within a defined cellular or organismal context. In this way, the tab complements the Protein Query interface: whereas the Protein Query tab is primarily used to retrieve protein-level responses associated with a selected compound, the Compound Query tab allows users to move from a protein of interest to the set of compounds linked to it across the available datasets. This functionality is particularly useful for candidate repurposing-oriented exploration and follow-up.

Similar to the Cell Line Query tab, this interface includes a robust suite of analytical tools, including filtering interactions by *p*-values, accessing UniProt protein descriptions, performing external checks with UniProt, and verifying drug-target interactions through external databases. Additionally, we provided the same functionality mentioned in the Cell Line Query tab for protein melting points, allowing users to access insights into melting point diversity across different cell lines and contexts.

### Protein Set Enrichment Tab

To enhance functional insights, the platform includes built-in tools for gene set enrichment analysis and singular enrichment analysis based on Gene Ontology (GO) annotation (48). These tools enable the investigation of potential shared functional characteristics among proteins associated with specific compounds. The analysis is rooted in the hypothesis that proteins targeted by a given chemical may share molecular functions, biological processes, or cellular compartments as defined by GO terms. The results of enrichment analyses are visualized through treemaps and network diagrams, offering intuitive representations of interrelated GO terms with similar semantic features.

This framework further supports the hypothesis that compounds targeting distinct protein groups may exhibit functional similarities, reflected in shared gene ontologies. By incorporating semantic similarity tools, users can quantitatively assess and visualize these relationships, providing valuable insights into the functional and mechanistic overlap among drug targets.

### Database Implementation

**Fig. 1:**
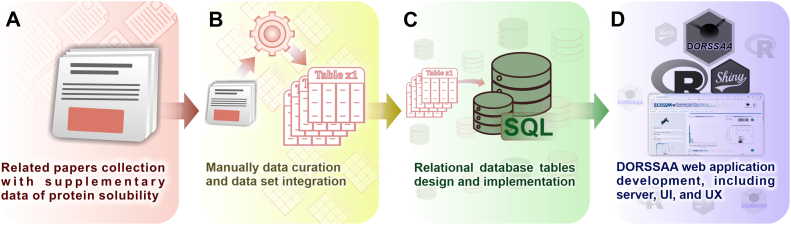
**Schematic representation of the DORSSAA workflow.***A,* data collection from relevant publications. *B,* manual data curation and integration into datasets. *C,* relational database schema design. *D,* implementation and development of the DORSSAA web application.

The DORSSAA backend server was developed using the R programming language (v.4.5.1, https://dorssaa.it.helsinki.fi/) in conjunction with the Shiny App framework to provide an interactive web-based user interface. SQL database functionality was integrated via the DBI R package, enabling efficient data management and querying. JavaScript code was incorporated on both the front-end and server sides to enhance functionality and interactivity. Statistical analyses and data processing were conducted entirely in R. DORSSAA was rigorously tested for compatibility and performance across multiple widely-used web browsers, including Google Chrome (preferred), Firefox, and Apple Safari. The system ensures seamless user interaction regardless of the chosen platform. The design and development workflow for DORSSAA is illustrated in Figure 1.

### Database Content

DORSSAA contains a comprehensive and diverse dataset tailored to the needs of researchers studying drug-protein interactions (Table 2):

A. **Number of Records**: The core Drug-Protein Interaction table includes 1,135,985 entries, representing interactions derived from 38 distinct cell lines and organisms, 135 compounds, and 40,742 proteins (as of 25 October 2025). The resource includes both unique and overlapping compound–context coverage. Specifically, some compounds were profiled across multiple biological contexts, whereas some cell lines or organisms were tested with multiple compounds, enabling users to examine both recurrence and breadth of profiling across the integrated studies.
B. **Data Accessibility**: Researchers can interact with the data through the DORSSAA web application, an intuitive and interactive web-based interface available at https://dorssaa.it.helsinki.fi/. The interface allows users to retrieve results for any drug-target interaction based on proteome solubility assay data. In addition, the current online version includes an Upload Data function that allows users to download a standardized submission template and contribute new datasets for manual review and future incorporation into DORSSAA.
C. **Ongoing Updates**: The database is updated regularly as new publications with publicly available TPP datasets are released. This ensures that DORSSAA remains a current and reliable resource for the scientific community.
D. **Long-term Availability**: The graphical user interface offers free access to researchers and is committed to indefinite availability, making DORSSAA a dependable and enduring tool for drug discovery and development investigations.

### Web Interface

**Fig. 2:**
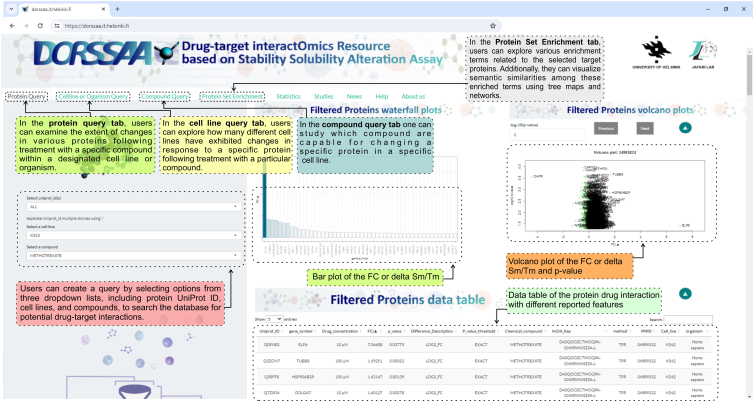
**The DORSSAA web application showcases an intuitive interface divided into three primary tabs: Protein Query, Cell Line Query, and Compound Query.** Users can initiate queries by selecting options from dropdown menus for UniProt_IDs, cell lines, and compounds. The main panel features visualizations such as bar charts and volcano plots, offering insights into drug-target interactions and protein-level changes. Below the visualizations, a detailed data table provides comprehensive information on all identified interactions, supporting efficient data exploration and in-depth analysis.

The DORSSAA web application offers an intuitive and interactive platform for data retrieval, visualization, and export (Fig. 2). The interface is structured around three main functional tabs: Protein Query, Cell Line Query, and Compound Query backed by additional analysis and utility views including Protein Set Enrichment, Statistics, Studies, News, Help, Upload Data, and About us.

To help users understand the extent and structure of the integrated resource, DORSSAA also provides coverage-oriented views summarizing how compounds and biological contexts are represented across studies, together with a Statistics-tab overlap table that makes repeated compound–context coverage explicit.

Users can initiate their queries by selecting options from three drop-down menus: UniProt_ID or Gene_symbol, Cell Line, and Compound. Upon selection, the main panel renders a bar chart and a volcano plot, and a detailed table summarizes all relevant interactions with comprehensive annotations and metrics (Fig. 2). These visual and tabular outputs let users examine drug-target interactions, assess protein-level changes, and extract insights into molecular mechanisms. In addition, the Statistics tab now includes an interactive overlap summary table that explicitly reports redundancy across compound–context coverage, including contexts tested by multiple compounds and compounds tested in multiple biological contexts. This helps users identify where DORSSAA supports cross-context profiling, repeated compound–context coverage, and broader comparative exploration across the database. In the current version, the query result tables also display the treatment context associated with the underlying study metadata, indicating whether the evidence was derived from intact-cell treatment, lysate/extract treatment, or both where applicable. Because this field is included directly in the interactive data tables, users can also search and filter the displayed results by treatment context during query exploration.

The Upload Data tab enables external users to contribute new datasets to DORSSAA through a guided submission workflow. Users can first download a standardized Excel template, complete it with the required study information and interaction data, and then upload the file through the web interface using the file-browse and submission options. To support curation, submitters are required to provide an academic email address together with additional metadata and a short message. After submission, the interface notifies users that the dataset will be reviewed by the DORSSAA team as soon as possible before potential integration into the resource. This functionality extends DORSSAA from a query-only platform into a growing community resource for curated data contribution.

Importantly, DORSSAA has undergone regular versioned releases to expand data coverage and functionality and to improve usability. Notable milestones include the addition of Cell line/organism search (v2.1.2), Compound Query and gene set enrichment analysis (v2.2.1), Meltome integration (v2.2.2), zebrafish and rat datasets (v2.2.3), FDR filtering and new datasets (v4.1.1), and a new database-search graphical user interface in the Statistics page with broader UI/Server upgrades plus updated Help/About (v4.1.2). Performance-focused updates (v3.1.1–3.2.1) optimized SQL/server layers and streamlined drop-downs for large queries. This active maintenance and roadmap reflect our commitment to making DORSSAA the primary resource for stability/solubility-based interactomics. *(See Release History Table for full version notes in the News tab)*.

## Results

### Case Study 1: a Proof-of-Principle Retrieval of the Canonical Methotrexate-Dihydrofolate Reductase Interaction Using DORSSAA

To illustrate the practical use of DORSSAA with a well-established reference interaction, we first queried methotrexate-associated proteins in A-549 cells using a single-study dataset derived from the cell-lysate condition.

We selected this example as a proof-of-principle case study because the methotrexate-Dihydrofolate Reductase (DHFR) interaction is a canonical and independently validated drug-target relationship. In this example, the initial retrieval step is based on one defined dataset rather than on pooled multi-study discovery; the added value of DORSSAA lies in enabling immediate follow-up across compounds, annotations, enrichment results, and additional studies from the same interface. Applying an FDR threshold of 0.05, we then examined how DORSSAA retrieves, ranks, and contextualize this interaction within the harmonized database.

Using DORSSAA’s Protein Query tool, we first retrieved methotrexate-associated proteins from the selected A-549 cell-lysate dataset and then used the broader DORSSAA framework to contextualize this canonical interaction across additional compounds and studies. In this context, the value of DORSSAA lies not in re-identifying DHFR as a novel target, but in enabling rapid target retrieval together with statistical and condition filtering, external cross-referencing, compound-centric follow-up, and functional interpretation within a single interface. GO enrichment of the methotrexate-associated proteins retrieved from this selected A-549 cell-lysate dataset highlighted six functional categories, including oxidative-stress-related biological processes, illustrating how DORSSAA can support biological interpretation at the single-context retrieval stage.

**Fig. 3:**
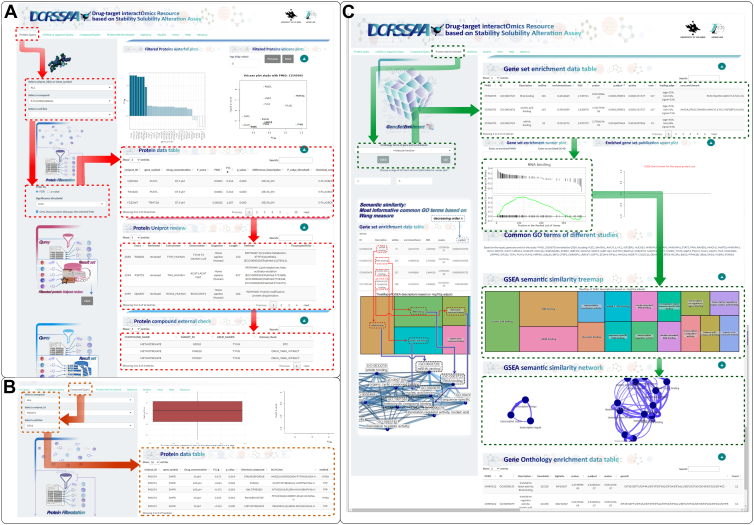
**Proof-of-principle retrieval and contextualization of the canonical methotrexate-DHFR interaction using DORSSAA.***A,* protein Query analysis identified methotrexate-associated proteins, with DHFR showing the strongest signal in the queried A-549 dataset. *B,* the Compound Query tab shows additional compounds linked to DHFR across studies, illustrating compound-centric exploration. *C,* enrichment and cross-study views place the canonical methotrexate-DHFR interaction in a broader biological and dataset context. Candidate additional associations are hypothesis-generating and were not independently validated in the current study. DHFR, Dihydrofolate Reductase

Among the 15 target records identified, DHFR (UniProt ID: P00374) emerged as a top target. DHFR exhibited the highest log2-fold change, with a highly significant FDR, underscoring its strong association with methotrexate in A-549 cells (Fig. 3*A*). DHFR is the cognate target of methotrexate. DHFR is known for its role in the biosynthesis of amino acids and folic acid, reducing dihydrofolate to tetrahydrofolate via the nicotinamide dinucleotide phosphate cofactor. Its therapeutic relevance in cancer and bacterial infections is well-documented. In DORSSAA, DHFR was also linked to five additional compounds across different studies, including staurosporine, SNS032, raltitrexed, and panobinostat, as shown in the Compound Query tab (Fig. 3*B*). This illustrates the compound-centric exploration capability of the platform rather than constituting validation of new therapeutic opportunities.

To further contextualize the methotrexate–DHFR interaction, enrichment analysis of the selected A-549 cell-lysate dataset highlighted biologically relevant terms, including processes related to transmethylation. The same canonical methotrexate–DHFR interaction could also be traced across additional datasets included in DORSSAA, notably the K562 TPP study (23) and the 293T STPP study (28), showing how the resource supports cross-study reproduction of known target relationships within related thermal-shift assay settings. In this example, the cross-study value of DORSSAA lies in this contextual follow-up across datasets rather than in a pooled multi-study GO enrichment analysis. Because the present case study was designed as a proof-of-principle demonstration, additional methotrexate-associated proteins surfaced by the platform are presented here as candidate associations for future investigation rather than validated off-targets. Overall, this example demonstrates DORSSAA’s utility for retrieval, contextualization, and prioritization of known and candidate interactions within a unified interface (Fig. 3*C*). This case study therefore illustrates a two-step use case for DORSSAA: single-context retrieval followed by integrated cross-study and cross-compound contextualization.

### Case Study 2: Drug-Protein Interactions in Molm16 Cells Investigated Using DORSSAA

A subset of DORSSAA directly derives from our recent combinatorial therapy study in 4 AML cell lines and includes 4 single drugs and 2 drug combinations (20, 21). Accordingly, the combinatorial conditions discussed below were not newly inferred by DORSSAA but were already part of the included AML dataset; the role of the resource here is to enable their integrated exploration from a protein-, compound-, and cell-context perspective. In this case study, we used DORSSAA to retrieve and contextualize context-specific protein-shift patterns across single-agent and combination conditions already represented within the integrated AML dataset.

**Fig. 4:**
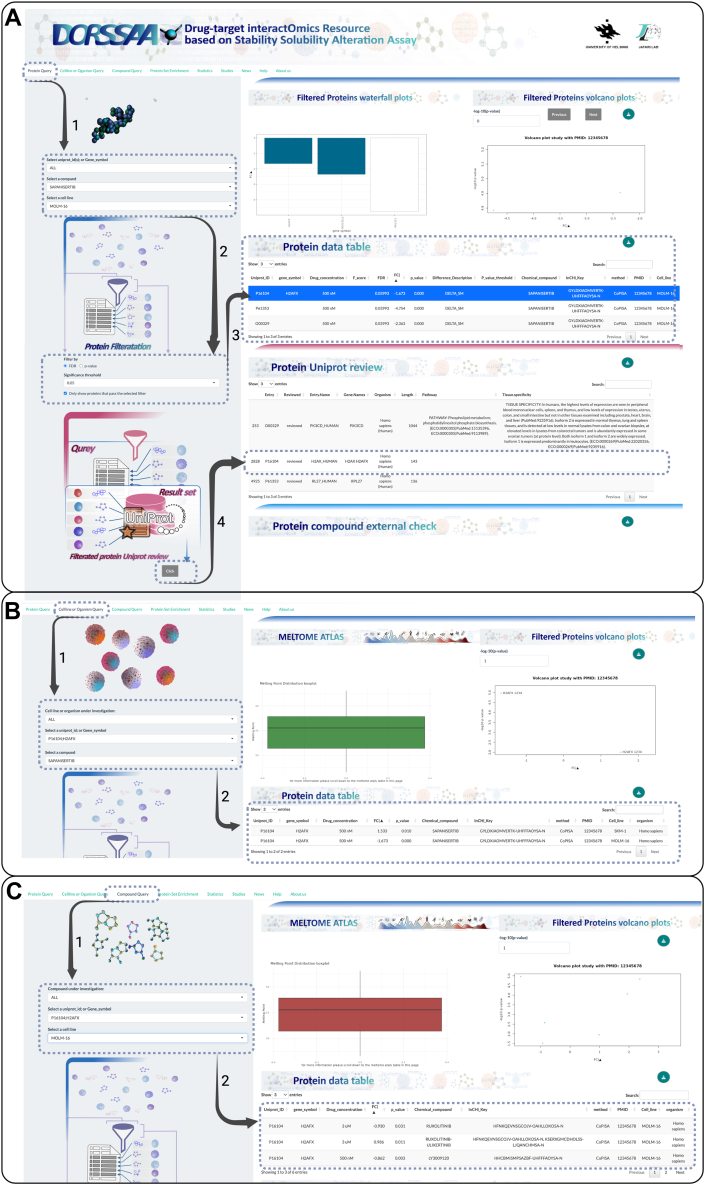
**Context-dependent H2AFX stability shifts in AML cell lines visualized using DORSSAA.***A,* in MOLM-16 cells, sapanisertib was associated with a significant H2AFX stability shift (UniProt P16104; log_2_ fold change = −1.673; FDR = 0.03993). *B,* in SKM-1 cells, a significant H2AFX shift was also observed with sapanisertib (log_2_ fold change = +1.533; *p* = 0.010). *C,* in MOLM-16, H2AFX-associated shifts were also observed for other single agents and combinations, including Ruxolitinib-Ulixertinib, LY3009120, Ruxolitinib, and Ulixertinib, each with distinct fold-change and significance values. These patterns are presented as pathway-level, hypothesis-generating observations rather than definitive evidence of direct target engagement.

To begin, we analyzed the effects of sapanisertib on the Molm16 cell line. In the Protein Query tab, significant targets were identified by applying an FDR threshold of 0.05. Among the resulting proteins, H2AFX (UniProt ID: P16104) stood out with a log-fold change of −1.673 and an FDR of 0.03993, indicating a significant sapanisertib-associated stability shift relative to untreated controls in an intact-cell context (Fig. 4*A*).

Because H2AFX is a chromatin-associated signaling readout, the observed stability shifts are more appropriately interpreted as possible consequences of upstream pathway perturbation than as direct evidence of H2AFX binding. In particular, kinase signaling changes may converge on DNA damage response regulators, including ATM, ATR, and DNA-PK, which influence H2AFX phosphorylation and chromatin-associated behavior. Thus, in the present case study, H2AFX is used as a hypothesis-generating marker of pathway-level perturbation rather than definitive direct target-engagement readout.

In the Cell Line/Organism Query tab, we assessed cell-type specificity across lines and cancer subtypes. Within the AML-focused subset analyzed here, the H2AFX-associated shift was significant only in MOLM-16 and SKM-1, indicating a cell-line-specific pattern among the four AML cell lines examined (Fig. 4B). In the Compound Query tab, additional agents associated with H2AFX shifts emerged, including Ruxolitinib-Ulixertinib, LY3009120, Ruxolitinib, and Ulixertinib, with varying log_2_ fold changes and *p*-values. Within the queried context, the Ruxolitinib + Ulixertinib combination was associated with a comparatively larger observed H2AFX shift (log_2_ fold change = 0.986; *p* = 0.011) (Fig. 4C); however, this observation is presented as a descriptive within-resource pattern rather than as a formal cross-study quantitative comparison. Here, the compared observations arise from a shared study context, but even within that setting, they are interpreted cautiously and not as definitive evidence of a stronger biological effect. Collectively, DORSSAA’s integrated views enable researchers to traverse targets, cell contexts, and both single-agent and combination conditions already present in the database, thereby supporting comparative interpretation and mechanistic hypothesis generation from context-dependent stability patterns (49, 50). In this example, the mixed H2AFX stabilization and destabilization patterns are consistent with perturbation of upstream signaling pathways (51), potentially including DNA damage response regulators that influence H2AFX phosphorylation, but they do not by themselves establish direct target engagement of H2AFX or of large upstream kinases. Accordingly, this case study is best interpreted as a hypothesis-generating illustration of pathway-level contextualization within DORSSAA that warrants orthogonal experimental validation (52).

### Case Study 3: Practical Use of DORSSAA for Exploratory Follow-Up of SCIN

Following a presentation of DORSSAA, a senior researcher interested in the protein SCIN (UniProt: Q9Y6U3) in diabetes asked whether the resource could help identify candidate compounds and informative experimental contexts for follow-up work. Although DORSSAA did not contain a diabetes-specific context for this protein, it enabled a structured exploratory workflow. Using the Protein Query tab, we confirmed that the queried UniProt entry corresponded to the reviewed protein SCIN and identified the contexts and compounds in which SCIN-associated shifts had been observed. The strongest available evidence was found in the K562 context, where multiple compounds were linked to SCIN, including sotrastaurin as one of the most statistically prominent hits.

We then used the Cell Line/Organism Query tab to assess whether other informative contexts were available and found that K562 represented the most useful context currently present in the database for this target. Finally, using the Compound Query tab and relaxing the significance threshold to *p* ≤ 0.05, we retrieved nine candidate compounds associated with SCIN in K562 (Table 3). In addition, the built-in Meltome layer in DORSSAA provided the available thermal-stability information for SCIN, allowing the researcher to inspect the range of reported melting points in the range of 45 to 50 °C for this protein across the Meltome available contexts. Together, these outputs provided a practical shortlist of compounds and a thermal-stability reference range that could help guide subsequent wet-lab validation and assay design. This example illustrates how DORSSAA can support real-world target-centered exploration by helping researchers move from a protein of interest to candidate compounds, available contexts, and complementary protein-stability information, even when the exact disease setting is not yet represented in the database.

**Table 3:** Candidate compounds associated with SCIN (Q9Y6U3) in the K562 context retrieved from DORSSAA at *p* ≤ 0.05, ranked by ascending *p*-value

Chemical compound	*p*-value	FC (fold change)
SOTRASTAURIN	0.005	−0.221
PRT062607	0.005	−0.224
VOLASERTIB	0.012	−0.197
RUCAPARIB	0.014	0.191
VORINOSTAT	0.025	0.173
ISPINESIB	0.030	−0.168
SGI-1776	0.032	−0.165
BS-181	0.042	−0.157
GEFITINIB	0.049	−0.149

## Discussion

DORSSAA is a dedicated resource for stability/solubility-based drug-target interactomics. By integrating advanced proteomics techniques, namely TPP and PISA, DORSSAA harnesses the power of mass spectrometry-based proteomics to characterize drug-protein interactions with high resolution and biological relevance.

Unlike transcript-only proxies, DORSSAA provides assay-aware, protein-level evidence to address critical questions in drug-target interactomics. These include: (i) Context-specific MoA mapping, identifying proteins stabilized or destabilized by compounds in defined cellular or organismal contexts; (ii) Off-target deconvolution, detecting non-canonical targets that shift consistently across studies; (iii) Target-centric querying, finding compounds that reproducibly modulate specific proteins or protein families across models; (iv) Cross-study replication, evaluating whether effect directions and magnitudes are consistent across independent assays and batches; (v) Combination design, distinguishing protein shifts unique to drug pairs versus single agents; (vi) Translational prioritization, identifying candidates that meet stringent FDR criteria, replicate across datasets, and map to tractable biology for follow-up; (vii) Generalizability vs. subtype specificity, determining whether target-compound interactions are broadly conserved or restricted to specific cancer subtypes; and (viii) Drug mimicry and repurposing, discovering compounds that recapitulate a target-compound’s thermal stability signature and may be rationally repurposed.

The core objective of DORSSAA is to accelerate therapeutic innovation and deepen our understanding of protein behavior under diverse conditions. Its extensive database compiles differential proteome solubility datasets from a wide range of sources, offering a rich and diverse proteomic landscape for exploration. As illustrated by the methotrexate-DHFR example, DORSSAA is particularly useful for retrieving canonical interactions and placing them in a broader compound-, context-, and enrichment-level framework, while any newly surfaced associations should be considered hypothesis-generating until independently validated.

A current limitation is that, although DORSSAA harmonizes outputs from multiple thermal and solubility-shift assays into a common searchable framework, the present study does not constitute a formal cross-assay benchmarking analysis. Differences in assay chemistry, sample type, temperature regime, compound exposure, proteome depth, and statistical processing may influence whether a target is detected, how strongly it ranks, and whether it is reproduced across studies. Accordingly, DORSSAA should presently be viewed as a harmonized exploration and hypothesis-generation resource rather than definitive evidence of full assay-to-assay comparability. This caution also applies to statistical interpretation: *p*-values and effect sizes displayed in DORSSAA are useful for within-study filtering and for exploratory cross-study contextualization, but they should not be treated as universally interchangeable quantitative measures across heterogeneous assays or statistical models. For the same reason, DORSSAA does not presently provide a cumulative global false-discovery rate across all integrated datasets. Instead, false-discovery control is maintained within study-local analysis families, and cross-study recurrence should be viewed as hypothesis-generating rather than as a single combined statistically controlled discovery space. A further interpretive consideration is that DORSSAA includes both lysate/extract-based and intact-cell datasets. Lysate/extract experiments are often more informative for candidate direct target nomination, whereas intact-cell datasets may also capture indirect downstream, pathway-level, or MoA-related consequences of compound treatment. For this reason, these evidence types should not be interpreted interchangeably, and observations from intact-cell studies should not automatically be described as direct target identification without orthogonal validation.

In its current online version, DORSSAA also supports curated community data submission through an Upload Data tab, allowing external researchers to propose additional datasets for review and integration (53), thereby increasing the long-term utility and scalability of the resource.

One of DORSSAA’s key strengths lies in its ability to support both focused and broad biological inquiries. This is further supported by the presence of both unique and overlapping compound-context coverage across the database, allowing users to ask not only whether a protein shift is observed in a single setting but also whether the same compound has been profiled across multiple contexts or whether a given biological context has been tested with multiple compounds. Researchers can investigate how specific proteins respond to compound treatments across various cell lines and organisms, while also uncovering broader patterns of proteomic change under different experimental contexts. The compound query functionality further enables identification of drugs that may affect particular proteins, supporting drug repurposing and the design of combination therapies.

By providing orthogonal, assay-native signals, DORSSAA complements large-scale perturbation screens and refines cancer-dependency discovery (1). It also informs the design and prioritization of synthetic-lethal strategies (2), offering a valuable layer of evidence for translational research.

Technically, DORSSAA is built on a robust foundation using R programming, SQL database functions, and JavaScript, ensuring a seamless and reliable user experience across major web browsers. The database currently includes data from 38 distinct cell lines and organisms, 135 unique compounds, and 40,742 protein targets, making it a highly versatile and expandable resource.

To remain relevant in the fast-evolving landscape of proteomics and drug discovery, DORSSAA is committed to continuous updates. By promptly integrating newly published, publicly available datasets, it ensures that users have access to the most current and comprehensive information. This dedication to staying current reinforces DORSSAA’s role as an essential tool for researchers tackling complex challenges in drug discovery and protein science.

## Data Availability

The script and data are publicly available at https://version.helsinki.fi/ehzange/dorssaa_19012024.

## Conflict of interest

The authors declare no competing of interests.
